# The value of contrast-enhanced ultrasonography in differential diagnosis of primary testicular germ cell tumors and non-germ cell tumors over 50 years old

**DOI:** 10.3389/fonc.2023.1090823

**Published:** 2023-02-20

**Authors:** Nianyu Xue, Guoyao Wang, Shengmin Zhang, Yijun Lu

**Affiliations:** ^1^ Department of Ultrasonography, Ningbo First Hospital, Ningbo, China; ^2^ Department of Urology, Ningbo First Hospital, Ningbo, Zhejiang, China; ^3^ School of Population and Public Health, University of British Columbia, Vancouver, BC, Canada

**Keywords:** ultrasonography, contrast-enhanced ultrasonography, testis, germ cell tumors, non-germ cell tumors

## Abstract

**Background:**

Unlike young and middle-aged patients, seminoma is not common in patients with primary testicular tumors over the age of 50, so it cannot follow the general ideas and norms for diagnosing and treating testicular tumors, and its characteristics need to be considered separately.

**Methods:**

The conventional ultrasonography and contrast-enhanced ultrasonography (CEUS) findings of primary testicular tumors in patients over 50 years old were retrospectively analyzed and compared with the pathological results to compare the diagnostic value of these two methods.

**Results:**

Of the 13 primary testicular tumors, 8 were primary lymphomas. Conventional ultrasound of 13 cases of testicular tumors showed hypoechoic with rich blood flow, and it was difficult to identify the type accurately. The sensitivity, specificity, positive predictive value, negative predictive value and accuracy of conventional ultrasonography in diagnosing non-germ cell tumors (lymphoma and Leydig cell tumor) were 40.0%, 33.3%, 66.7%, 14.3%, and 38.5%, respectively. CEUS findings: 7 of 8 lymphomas showed uniform hyperenhancement. 2 cases of Leydig cell tumors showed uniform high enhancement. 2 cases of seminoma and 1 case of spermatocytic tumor showed heterogeneous enhancement, with necrosis in the interior. The sensitivity, specificity, positive predictive value, negative predictive value and accuracy rate of non-germ cell tumor diagnosis according to the non-necrotic area of CEUS were 90.0%, 100.0%, 100.0%, 75.0% and 92.3%, respectively. Compared with conventional ultrasound, the difference was statistically significant (P=0.039).

**Conclusions:**

Primary testicular tumors in patients over 50 years old are mainly lymphoma, and CEUS is significantly different between germ cell tumors and non-germ cell tumors. Compared with conventional ultrasound, CEUS can distinguish testicular germ cell tumors from non-germ cell tumors more accurately. Preoperative ultrasonography is significant for accurate diagnosis and can guide clinical treatment.

## Introduction

Testicular tumors account for 1-2% of male malignant tumors (1). The most common malignant tumors in adolescent males are divided into primary tumors and secondary tumors. Due to the existence of primary lesions and systemic manifestations, secondary tumors are Relatively easy to diagnose. Although there are many pathological types of testicular tumors, most of them are germ cell tumors (2, 3). Ultrasound, the first-choice imaging examination for scrotal disease (3, 4), is easy to detect masses, but qualitative diagnosis is more difficult. Since most testicular tumors are malignant, seminoma is the most common (5), and needle biopsy can lead to local recurrence. Therefore, a needle biopsy is generally not recommended for testicular tumors. For testicular tumors smaller than 15 mm, the testicles can be preserved in conjunction with intraoperative frozen sections (6, 7). However, this inevitably leads to a longer surgery time. While for large lesions >4 cm, even if lymphoma is suspected, radical orchiectomy is mandatory in most of cases to relieve symptoms and reduce tumor bulk. However, if lymphoma is diagnosed preoperatively by ultrasound, a puncture biopsy can be taken to make a definitive diagnosis, thus saving time for surgery. However, seminoma is uncommon in elderly patients (2), so it cannot follow the general ideas and norms for diagnosing and treating testicular tumors, and its characteristics need to be considered separately. This study retrospectively analyzed the conventional ultrasonography and contrast-enhanced ultrasonography of primary testicular tumors in patients over 50 years old, in order to make an accurate preoperative diagnosis and guide clinical treatment.

## Methods

### Subjects

This retrospective study was approved by the Ethics Committee of The First Ningbo Hospital (2021RS105). A retrospective analysis of 14 cases of primary testicular tumors confirmed by pathology in our hospital from January 2013 to December 2021. Inclusion criteria: (1) confirmed by histopathology; (2) with complete routine ultrasound and CEUS data; (3) age > 50 years. Exclusion criteria: (1) incomplete data: lack of histological and pathological results, incomplete ultrasound images; (2) received non-steroidal anti-inflammatory drugs, radiotherapy, chemotherapy and other immunotherapy; (3) Elevated AFP or HCG. The age ranged from 51 to 67 years old, with an average of (59.6 ± 5.3) years old. According to the pathological results, they were divided into non-germ cell tumor group (lymphoma and Leydig cell tumor) and germ cell tumor group (seminoma and spermatogenic tumor).

### Equipment and agents

The contrast agent used in CEUS was SonoVue (Bracco SpA, Milan, Italy). The agents were microbubbles of the phospholipids microencapsulated sulfur hexafluoride (SF 6). The microbubbles had an average diameter of 2.5 μm and pH values of 4.5–7.5. After the SonoVue powder was thoroughly dissolved in 5 mL of normal saline, 2.4 mL of the solution was injected into the bolus through the cubital vein.

The ultrasound devices used included the Aplio500 (TOSHIBA CORPORATION, Tokyo, Japan), LOGIQ E9 GE (General Electric Company, Boston, Massachusetts, USA), EPIQ7 (Philips Electronic N.V, Amsterdam, The Netherlands), EUB-8500 (HITACHI, Tokyo, Japan), and Aixplorer (SuperSonic Imagine, Aix-en-Provence, France). The CEUS function was available on all of these devices. A linear array probe was used (frequency 5.0 -12.0 MHz).

### Methodology

The conventional ultrasound and CEUS images of the primary testicular tumors were retrospectively analyzed. The parameters of the conventional ultrasound images included the location, number, size, shape, echo, boundary, and blood flow. The peripheral annular blood flow was used as the diagnostic criterion for Leydig cell tumors and the perforating vessel was used as the diagnostic criteria for lymphoma. If the testicular tumor is hypoechoic with abundant blood flow and irregular blood flow distribution, it is diagnosed as germ cell tumor. The findings were interpreted by 2 physicians with 10 years of experience in scrotal ultrasound, and each preliminary diagnosis was made after the physicians reached an agreement. The parameters of CEUS included the enhancement time, enhancement level (high, equal, low, or none), and contrast-agent distribution (uniform or non-uniform). CEUS showing uniform high enhancement was used as the diagnostic criterion for non-germ cell tumor (lymphoma and Leydig cell tumor). CEUS showing heterogeneous enhancement with necrosis in the interior was used as the diagnostic criterion for germ cell tumors of the testis. The findings were interpreted by 2 physicians with 5 years of experience in scrotal ultrasound, and each preliminary diagnosis was made after the physicians reached an agreement. All 4 physicians were blind to the final diagnoses and other imaging information at the time of the interpretation and preliminary diagnoses.

**Figure 1:**
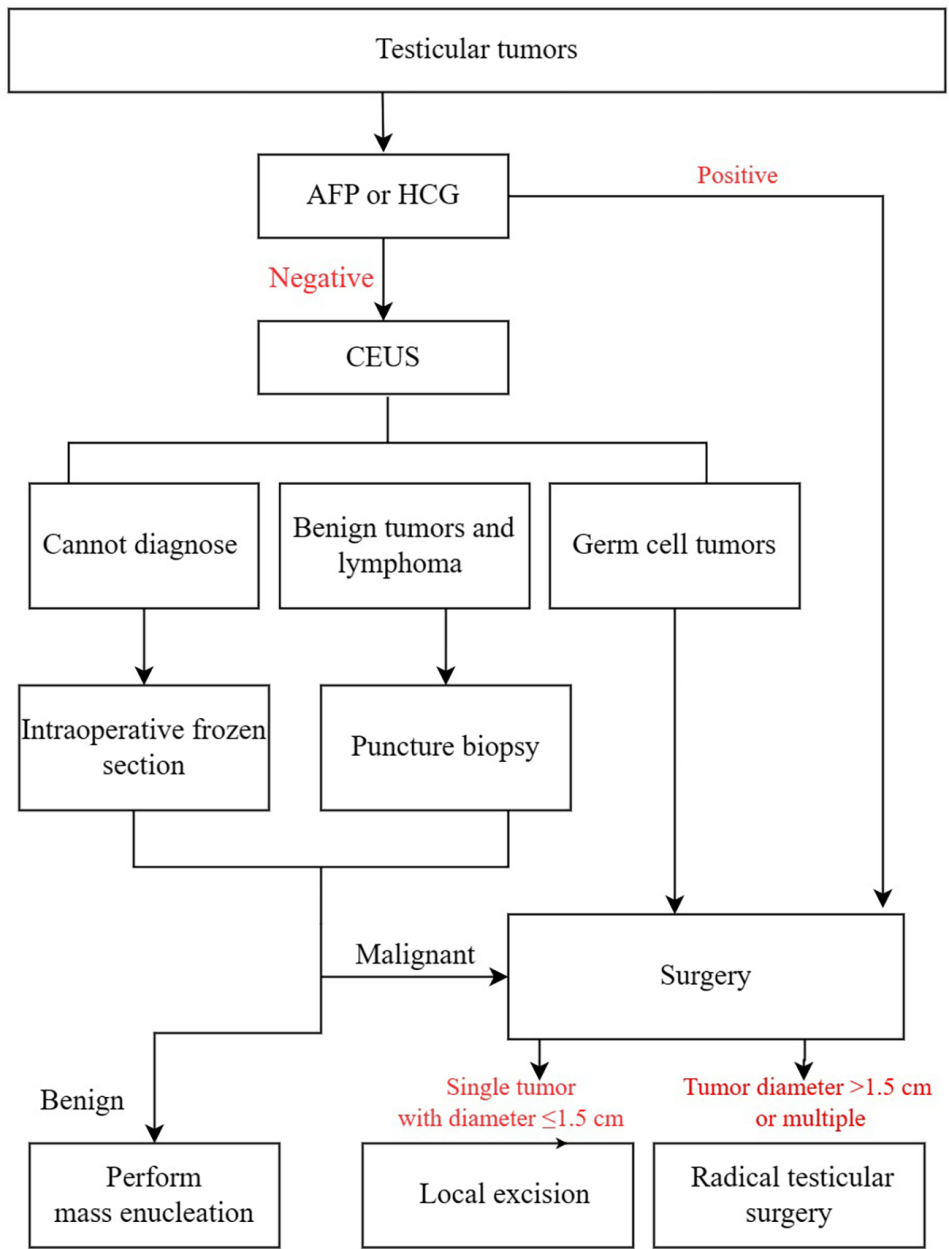
Clinical flow chart.

The clinical flow chart for the diagnosis and treatment of testicular tumors is shown in **Figure 1**.

### Statistical analysis

The statistical analysis was performed using the SPSS13.0 software package (Chicago, IL, USA). The count data were analyzed using the paired χ2 test, and the diagnostic accuracy of the examinations was assessed in 2-by-2 tables. The measurement data were expressed as the mean ± SD. A P value <0.05 was considered statically significant.

## Results

### Clinicopathological data

A total of 12 patients (13 masses in total), except for 1 case of bilateral testicular lymphoma, the others were unilateral and single. Among the 13 cases of primary testicular tumors confirmed by pathology, 10 were allocated to the non-germ cell tumor group(including 8 cases of primary lymphoma and 2 cases of Leydig cell tumors) and 3 were allocated to the germ cell tumor group(including 2 cases of seminomas, and 1 case of spermatocytic tumor). The lesions occurred in the left testis in 8 patients and in the right testis in 5 patients.

Except for 2 cases of Leydig cell tumors found by ultrasonography, the others were all due to scrotal enlargement (1 case of bilateral testicular lymphoma with scrotal pain, the rest were painless). 1 case of bilateral lymphoma had elevated follicle-stimulating hormone and luteinizing hormone, and no other related abnormalities were found.

### Findings of conventional ultrasound and CEUS

**Figure 2:**
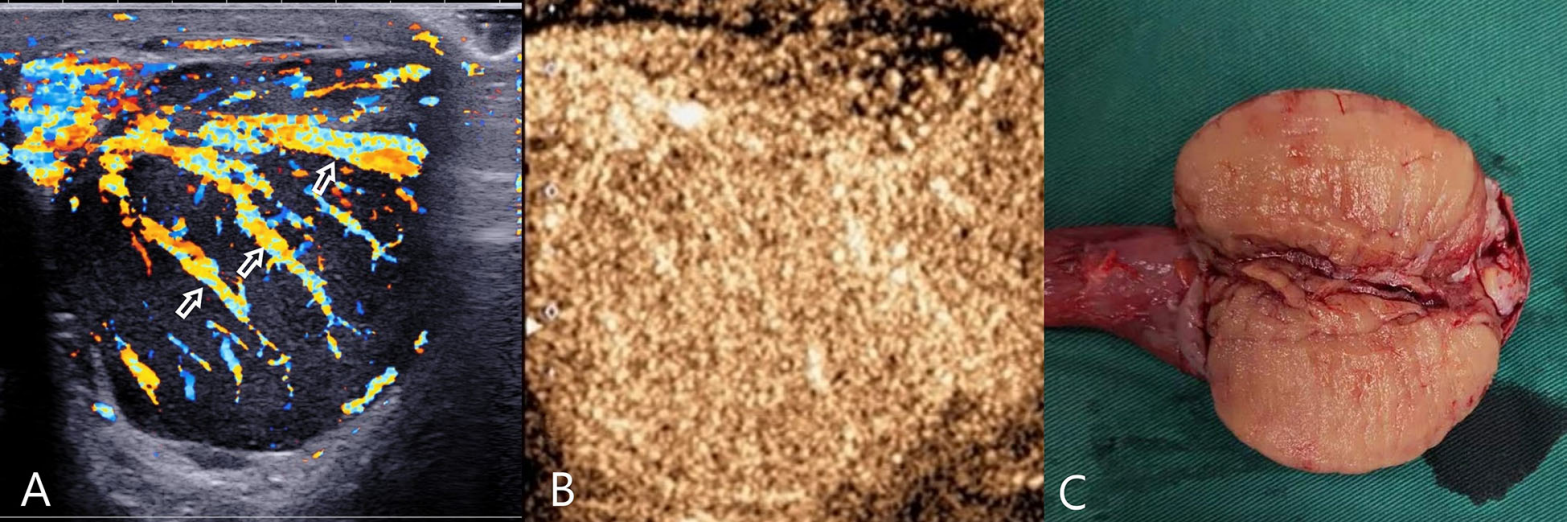
Testicular lymphoma. **(A)** CDFI: hypoechoic mass in the left testis, abundant blood flow, with multiple perforating vessels(arrow); **(B)** CEUS, CEUS showed uniform and high enhancement, and no obvious necrotic area was found. **(C)** Macro-section, Mass occupying the entire testicle; CDFI, Color Doppler Flow Imaging; CEUS, contrast-enhanced ultrasonography.

**Figure 3:**
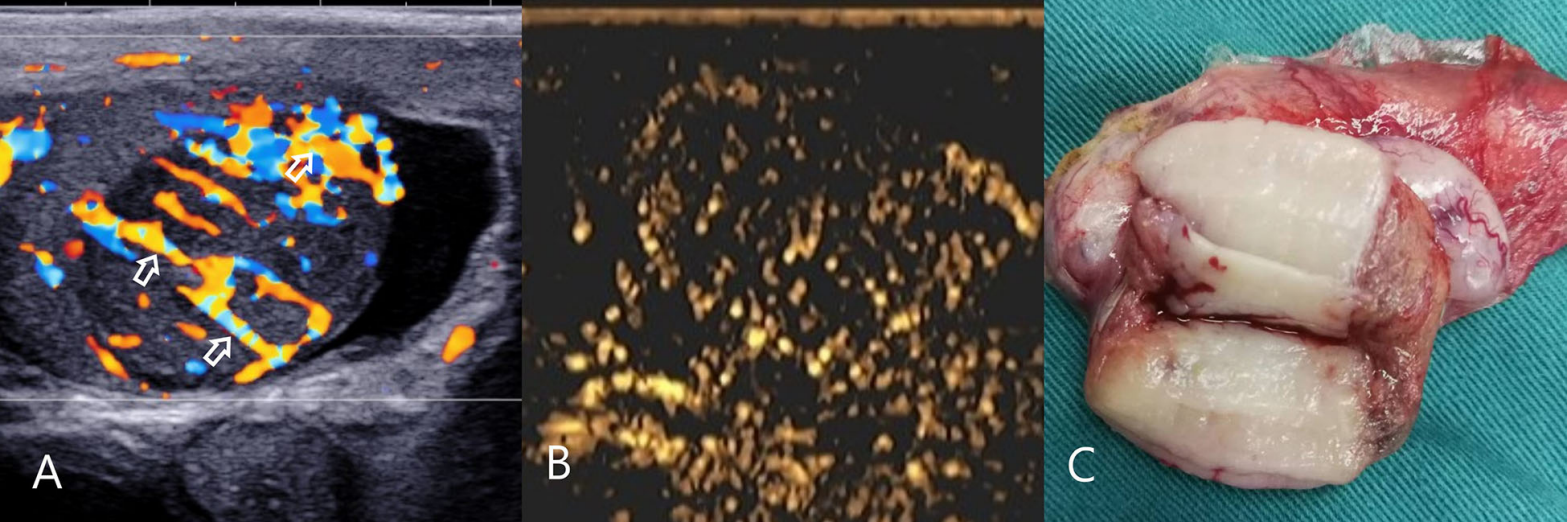
Testicular spermatocytic tumor. **(A)** CDFI: hypoechoic mass in the right testis, abundant blood flow and multiple perforating vessels(arrow), consistent with the ultrasound findings of lymphoma. **(B)** CEUS: The CEUS showed heterogeneous sparse and low enhancement, which was significantly different from that of lymphoma. **(C)** Macro-section, Mass occupies most of the testicle; CDFI, Color Doppler Flow Imaging; CEUS, contrast-enhanced ultrasonography.

**Figure 4:**
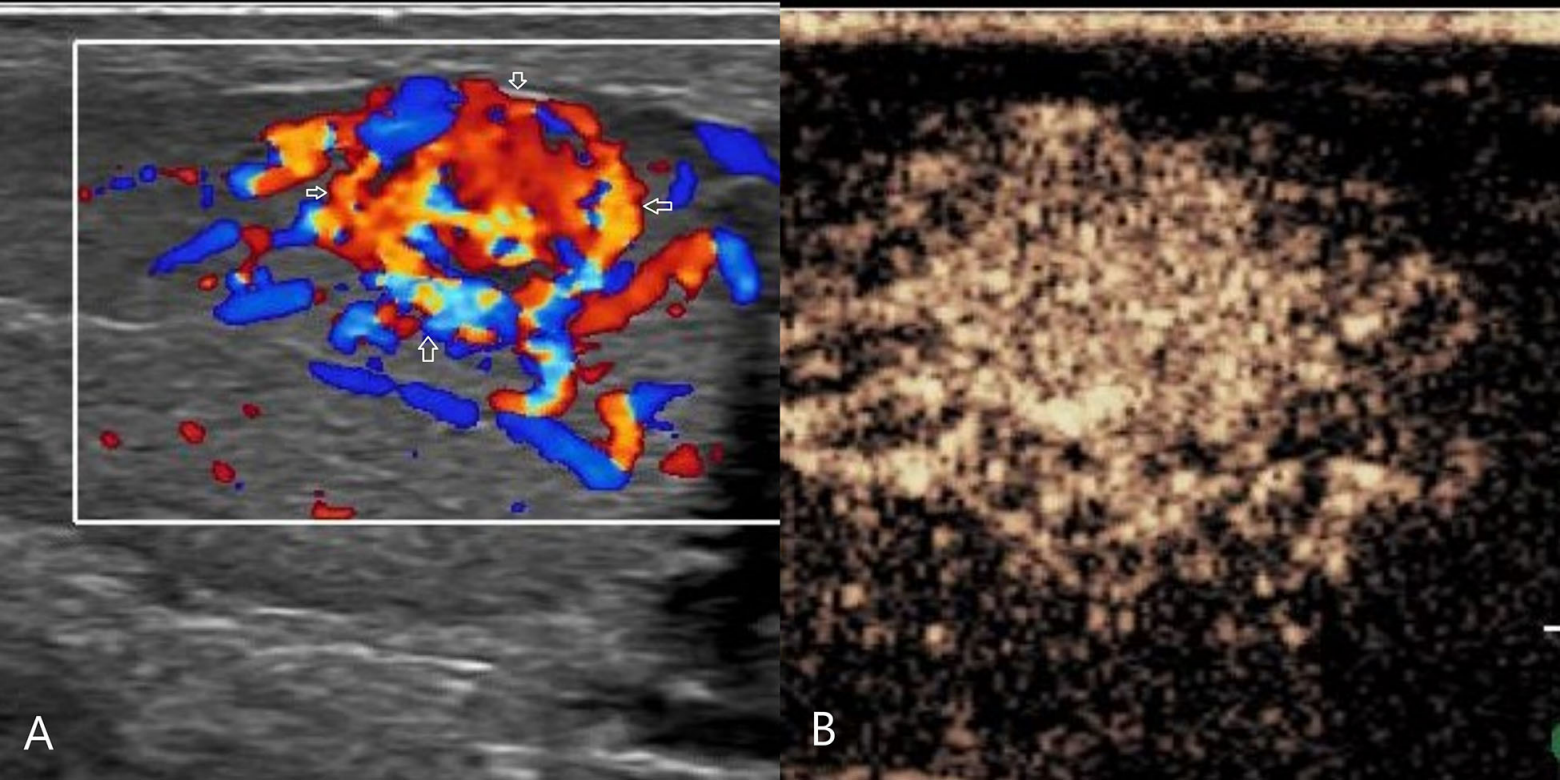
Leydig cell tumor. **(A)** CDFI: hypoechoic mass in the left testis, abundant blood flow, with annular blood flow around(arrow). **(B)** CEUS: uniform and high enhancement, and no obvious necrotic area was found. CDFI, Color Doppler Flow Imaging; CEUS, contrast-enhanced ultrasonography.

The characteristics of conventional ultrasound and CEUS in testicular germ cell tumors and non-germ cell tumors over 50 years old are shown in **Table 1**. All 13 showed abundant blood flow, including 3 lymphomas, 1 spermatocytic tumor, and 1 seminoma with perforating vessels (**Figures 2**, **3**), and 1 Leydig cell tumor with peripheral circular blood flow (**Figure 4**), other blood flow is irregular. The characteristics of conventional ultrasound in 13 cases were similar, and it was difficult to identify their pathological types accurately. The sensitivity, specificity, positive predictive value, negative predictive value and accuracy rate of conventional ultrasound in the diagnosis of non-germ cell tumors were respectively 40.0%, 33.3%, 66.7%, 14.3%, and 38.5%.

**Table 1:** Conventional ultrasound and CEUS features of testicular germ cell tumors and non-germ cell tumors over 50 years old.

Conventional ultrasound and CEUS	germ cell tumors group (n=3)	non-germ cell tumors group (n=10)
Age (years), mean ± SD	58.0 ± 7.5	60.1 ± 4.8
Lesion size (cm), mean ± SD	5.1 ± 2.4	4.2 ± 2.7
Boundary, n		
Clear	2	10
Unclear	1	0
Echoes, n		
Hypoechoic	3	10
Shape, n		
Round or oval	2	7
Irregular	2	3
Echo distribution,n		
Homogenous	0	4
Heterogenous	3	6
Blood flow, n		
Perforating vessels	2	3
Peripheral annular	0	1
Irregular	1	6
Enhancement, n		
Uniform hyperenhancement	0	1
Heterogeneous enhancement	3	9

SD, standard deviation; CEUS, contrast-enhanced ultrasonography.

CEUS: 8 cases of lymphoma showed fast forward and fast regression, 7 cases showed uniform high enhancement, no obvious necrosis area (**Figure 2**), 1 case of lymphoma showed obvious necrosis area; 2 cases of seminoma showed high enhancement around the periphery, with necrosis in the interior, showing fast forward and fast regression; 1 case of spermatocytic tumor showed uneven, sparse, low enhancement, fast forward and equal regression (**Figure 3**); 2 cases of Leydig cell tumors showed uniform high enhancement, no obvious necrotic area, and showed rapid progress and slow regression (**Figure 4**). Spermatocytic tumor was the only tumor with the low enhancement of all tumors. Lymphoma and Leydig cell tumor showed uniform high enhancement, lymphoma fast forward and fast regression, Leydig cell tumor fast forward and slow regression. The sensitivity, specificity, positive predictive value, negative predictive value and accuracy rate of non-germ cell tumor diagnosis according to the non-necrotic area of CEUS were 90.0%, 100.0%, 100.0%, 75.0% and 92.3%, respectively. Compared with conventional ultrasound, the difference was statistically significant (P=0.039).

## Discussion

The most common testicular tumor in adolescents is seminoma (5), so the choice of radical orchiectomy is most feasible. However, seminoma is very rare in patients over 50 years old. From this study, lymphoma is the main one. Since lymphoma is a combination treatment requiring surgery, chemotherapy and radiotherapy, intraoperative frozen sections are more difficult to diagnose, so preoperative puncture biopsy for immunohistochemistry can clarify the diagnosis (8). Since Leydig cell tumors are mostly benign, enucleation is the first choice (9, 10). Pathological diagnosis of Leydig cell tumors by preoperative biopsy can reduce the operation time and protect testicular tissue. Therefore, the correct diagnosis is of great significance to the patient and clinical, which also puts forward higher requirements for ultrasound diagnosis, not just the diagnosis of malignancy.

Relying on the history of the primary tumor and systemic manifestations, secondary testicular tumors are relatively easy to diagnose. Primary testicular tumors are more challenging to diagnose. This study shows that most of the primary testicular tumors are large. This is due to the relatively fast growth rate of lymphoma and embryonal carcinoma. In the early stage of the tumor, the tumor is small and generally has no clinical symptoms. Only when the tumor grows to a certain extent clinical symptoms are detected. Painless enlargement of the scrotum is the most common symptom in most patients (only Leydig cell tumors are occasionally found in the scrotum due to inguinal hernia), but this is also a common feature of testicular tumors and is not specific. This study showed that 61.5% were lymphoma, and there were only 2 cases of seminoma. Therefore, patients over 50 years old with painless scrotal enlargement should first consider lymphoma.

This study showed that primary testicular tumors had very similar gray-scale ultrasonographic appearances (most of them showed clear borders, round-like hypoechoic), and it was difficult to distinguish pathological types. Color Doppler blood flow has a certain value in diagnosing testicular tumors, and perforating vessels have a certain value in diagnosing lymphoma. The reason for the formation of perforating vessels may be: lymphoma is a disease mainly caused by single-cell proliferation, and the lesions originate in the interstitium of the testis, so the original vascular anatomy in the testis may not be affected, which is different from benign tumors on vascular compression, malignant tumors present differently to vascular compression and erosion. This study showed that perforating vessels appeared in 3 lymphoma masses, and the diagnosis was based on this. The remaining 5 lymphomas only showed abundant blood flow without perforating vessels. 1 case of spermatocytic tumor and 1 case of seminoma had perforating vessels, which were misdiagnosed as lymphoma by conventional ultrasonography. These perforating vessels may be elongated feeding vessels of the tumor. 1 case of Leydig cell tumor showed abundant blood flow with annular blood flow around it. This sign is not seen in other testicular tumors, so peripheral annular blood flow can be used as a specific sign of Leydig cell tumors (to be further confirmed in large sample studies).

In this study, CEUS can accurately distinguish germ cell tumors from non-germ cell tumors (lymphoma and stromal cell tumor) based on the presence or absence of necrosis, with an accuracy rate of 92.3%. Seminoma over the age of 50 is relatively rare. This study showed that the 2 cases of seminoma showed fast forward and fast regression, heterogeneous high enhancement, and necrotic areas. Combined with negative tumor markers, a diagnosis can be made. Spermatocytic tumor was the only tumor with low enhancement on CEUS of all tumors in this study, which can be diagnosed in combination with negative tumor markers. In the previous WHO classification, spermatocytic tumor was regarded as a subtype of seminoma, called spermatocytic seminoma (11). The 2016 WHO classification separates spermatocytic tumor from seminoma. Although the cytological appearance of these two tumors are similar (12), the CEUS appearance is significantly different. The CEUS of lymphomas all showed fast forward and fast backward, except for 1 case with necrosis. The others showed uniform hyperenhancement. Therefore, in patients over 50 years old, a painless testicular mass, perforating vessels, uniform high enhancement on CEUS, and negative tumor markers can be diagnosed as lymphoma. Leydig cell tumor of the testis generally has a small mass. The CEUS shows uniform high enhancement, fast forward and slow regression, which is different from lymphoma, and can make a clear diagnosis. Enucleation of the mass was performed to avoid the removal of the testis. Pathological diagnosis of Leydig cell tumors and lymphomas by preoperative biopsy can reduce the operation time and protect testicular tissue.

The present study had some limitations. First, this study is a retrospective analysis. The incidence of testicular tumors is low, the sample size is limited, and further research with large samples and multiple centers is needed. Second, limited by the retrospective design and the difference in match models, we did not perform a CEUS-based quantitative analysis. Third, because of the extremely high accuracy of the diagnosis of testicular tumors by the professional andrology sonographers in our center, they have won the trust of andrologists. Most testicular tumors have not undergone magnetic resonance examination, so there is no other image for comparative analysis.

In conclusion, the primary testicular tumors in patients over 50 years old are mainly lymphoma, and CEUS is significantly different between germ cell tumors and non-germ cell tumors. Compared with conventional ultrasound, CEUS can distinguish testicular germ cell tumors from non-germ cell tumors more accurately. Preoperative ultrasonography is of great significance for a clear diagnosis, which can guide clinical treatment and avoid unnecessary orchiectomy.

## Data availability statement

The raw data supporting the conclusions of this article will be made available by the authors, without undue reservation.

## Ethics statement

The studies involving human participants were reviewed and approved by Ethics Committee of The First Ningbo Hospital. Written informed consent for participation was not required for this study in accordance with the national legislation and the institutional requirements.

## Author contributions

Author Contributions Statement: All authors wrote and designed the main manuscript text, and reviewed the manuscript. NX is responsible for data collection and compilation. NX, GW and SZ are responsible for the analysis and interpretation of data and the final approval of manuscript. YL is responsible for the revision and guidance of language manuscripts. All authors contributed to the article and approved the submitted version.
